# Xylem cavitation resistance can be estimated based on time‐dependent rate of acoustic emissions

**DOI:** 10.1111/nph.13476

**Published:** 2015-05-22

**Authors:** Markus Nolf, Barbara Beikircher, Sabine Rosner, Anton Nolf, Stefan Mayr

**Affiliations:** ^1^ Institute of BotanyUniversity of InnsbruckSternwartestr. 15Innsbruck6020Austria; ^2^ Hawkesbury Institute for the EnvironmentUniversity of Western SydneyRichmondNSW2753Australia; ^3^ Institute of BotanyBOKU ViennaGregor Mendel Str. 33Vienna1180Austria; ^4^ Institute for Experimental PhysicsUniversity of InnsbruckTechnikerstr. 25Innsbruck6020Austria

**Keywords:** acoustic activity, acoustic emission (AE), drought stress, hydraulic vulnerability, plant–water relations, xylem

## Abstract

- Acoustic emission (AE) analysis allows nondestructive monitoring of embolism formation in plant xylem, but signal interpretation and agreement of acoustically measured hydraulic vulnerability with reference hydraulic techniques remain under debate.
- We compared the hydraulic vulnerability of 16 species and three crop tree cultivars using hydraulic flow measurements and acoustic emission monitoring, proposing the use of time‐dependent AE rates as a novel parameter for AE analysis.
- There was a linear correlation between the water potential (Ψ) at 50% loss of hydraulic conductivity (P_50_) and the Ψ at maximum AE activity (P_maxrate_), where species with lower P_50_ also had lower P_maxrate_ (*P *<* *0.001, *R* ^2^ = 0.76).
- Using AE rates instead of cumulative counts for AE analysis allows more efficient estimation of P_50_, while excluding problematic AE at late stages of dehydration.

## Introduction

Acoustic emissions (AE) have long been used to detect cavitation (Milburn & Johnson, 1966) and estimate hydraulic vulnerability to drought or freezing stress (e.g. Tyree *et al*., 1984; Tyree & Sperry, 1989; Hacke *et al*., 2000; Mayr *et al*., 2007; Mayr & Sperry, 2010; Cochard *et al*., 2013; Charrier *et al*., 2014). Cavitating xylem conduits undergo rapid tension release when water vapour replaces water under negative pressure, which produces AE at both audible and ultrasonic frequencies (Milburn & Johnson, 1966; Tyree & Dixon, 1983; Ritman & Milburn, 1988). Modern measurement systems allow automated monitoring of AE in the high‐frequency range and analysis of multiple parameters, such as amplitude, energy and waveform features, but additional signals from nonconducting sources make AE analysis more complex (Tyree & Dixon, 1986; Ritman & Milburn, 1988; Cochard & Tyree, 1990; Hacke & Sauter, 1996; Kikuta, 2003; Mayr & Rosner, 2011; Wolkerstorfer *et al*., 2012).

In some species, especially conifers, quantitative analysis of cumulative acoustic emissions as an indirect measure of drought‐induced embolism was well correlated with hydraulically measured conductivity loss (e.g. Lo Gullo & Salleo, 1993; Hacke & Sauter, 1995; Hacke *et al*., 2000; Johnson *et al*., 2012), and the acoustic energy released during cavitation reflected conduit dimensions and the intensity of drought stress (Mayr & Rosner, 2011; Ponomarenko *et al*., 2014). However, other species, including many angiosperms, have revealed varying offsets between hydraulically and acoustically analysed hydraulic failure (e.g. Tyree & Dixon, 1986; Jackson & Grace, 1996; Nardini *et al*., 2001; Manoharan & Pammenter, 2005; Johnson *et al*., 2009). A common phenomenon among this second group of species is the fact that acoustic activity does not cease when samples are fully embolized (e.g. Tyree & Dixon, 1986; Hacke & Sauter, 1996; Kowalski & Smoczkiewicz, 2004; Wolkerstorfer *et al*., 2012; Rosner, 2015). Possible causes for these additional signals may be emissions from nonconducting cells or tissues such as sclerenchyma (Kikuta & Richter, 2003) or bark (Kikuta, 2003). Cavitating fibres (Tyree & Dixon, 1986; Jackson & Grace, 1996), ray cells (Tyree & Sperry, 1989) or shrinkage processes and related microfracturing of wood have also been reported to produce AE (Kikuta & Richter, 2003; Kowalski & Smoczkiewicz, 2004; Wolkerstorfer *et al*., 2012; Vergeynst *et al*., 2014b).

Recent improvements in AE analysis were made using acoustic parameters such as absolute signal energy (e.g. Rosner *et al*., 2006; Johnson *et al*., 2009; Mayr & Rosner, 2011; Wolkerstorfer *et al*., 2012; Vergeynst *et al*., 2014a). Other authors have manually set arbitrary end points for AE recording (Hacke & Sauter, 1996; Kikuta *et al*., 1997; Salleo *et al*., 2001). However, signal interpretation and analysis remain challenging, and thus the use of acoustic methods for hydraulic vulnerability analyses has remained limited. Meanwhile, the accuracy of hydraulic methods, including the benchtop dehydration (Sperry *et al*., 1988) and centrifuge techniques (Cochard, 2002), is also currently under debate (Jansen *et al*., 2015), as measurement artefacts were shown for both methods, especially with long‐vesselled species (e.g. Beikircher *et al*., 2010; Cochard *et al*., 2010, 2013; Wheeler *et al*., 2013; Torres‐Ruiz *et al*., 2015).

In this study, we focused on the use of acoustic activity as a function of time, rather than absolute cumulative emission counts. We hypothesized that the highest acoustic activity should occur near the steepest part of a typical vulnerability curve, that is, near its inflection point (P_50_), when most embolism is forming within a narrow range of water potential (Ψ). Therefore, the Ψ at maximum AE activity should be correlated to a species’ hydraulically measured P_50_.

## Materials and Methods

The study was conducted on a total of 16 woody species. The dataset includes both published (Beikircher *et al*., 2013) and unpublished hydraulic data, covering five temperate tree species, six shrub species from a dry temperate inner‐alpine forest, three high‐yield apple cultivars, three tropical rainforest species, and one species of woody vine (Table 1). Hydraulic reference measurements and AE testing were performed on plant material from the same populations or, when possible, from the same plants.

**Table 1:** Study species and growth type, sampling location, hydraulic method and plant part used, and data source

Species	Growth type	Sampling location (nearest town/city)	Hydraulic method	Branch/shoot	Study
Angiosperms					
*Amelanchier ovalis*	Shrub	Innsbruck, AT	Cavitron	Branch	This work
*Berberis vulgaris*	Shrub	Innsbruck, AT	Cavitron	Branch	This work
*Dysoxylum papuanum*	Tree	Cape Tribulation, AU	Sperry	Branch	M. Nolf *et al*. (unpublished)
*Elaeocarpus grandis*	Tree	Cape Tribulation, AU	Sperry	Branch	M. Nolf *et al*. (unpublished)
*Hedera helix*	Vine	Innsbruck, AT	Sperry	Shoot	This work
*Lonicera xylosteum*	Shrub	Innsbruck, AT	Cavitron	Branch	This work
*Malus domestica* var. *Braeburn*	Tree	Latsch, IT	Sperry	Branch	Beikircher *et al*. (2013)
*Malus domestica* var. *Golden Delicious*	Tree	Latsch, IT	Sperry	Branch	Beikircher *et al*. (2013)
*Malus domestica* var. *Red Delicious*	Tree	Latsch, IT	Sperry	Branch	Beikircher *et al*. (2013)
*Populus alba*	Tree	Vienna, AT	Sperry	Shoot	This work
*Populus tremula*	Tree	Vienna, AT	Sperry	Shoot	This work
*Quercus petraea*	Tree	Vienna, AT	Sperry	Shoot	This work
*Sorbus aucuparia*	Tree	Vienna, AT	Sperry	Branch	This work
*Syzygium sayeri*	Tree	Cape Tribulation, AU	Sperry	Branch	M. Nolf *et al*. (unpublished)
*Viburnum lantana*	Shrub	Innsbruck, AT	Cavitron	Branch	This work
Gymnosperms					
*Juniperus communis*	Shrub	Innsbruck, AT	Cavitron	Branch	This work
*Picea abies*	Tree	Innsbruck, AT	Sperry	Branch	This work
*Pinus mugo*	Shrub	Innsbruck, AT	Cavitron	Branch	This work

Hydraulic methods: Cavitron (centrifuge technique; Cochard, 2002), Sperry (benchtop drying and measurement of flow before and after removal of embolism; Hietz *et al*., 2008; Sperry *et al*., 1988). Studies: Beikircher *et al*. (2013), M. Nolf *et al*. (unpublished). AT, Austria; AU, Australia; IT, Italy.

### Sample preparation and water potential determination

Long branches or shoots (typically 2–3 m long; Table 1) were collected from the field by cutting them under water (shrub species, apple cultivars), or cutting in air at full saturation early in the morning (tree species from Vienna, vine species) or before dawn (Australian rainforest species). All branches (sampled under tension and without tension) were immediately recut two to three times under water in the field, and one to two times in the laboratory, and rehydrated for at least 24 h while covered in plastic foil before benchtop drying or further sample preparation.

Water potential (Ψ) was measured on up to three unbagged leaves or small terminal branches on side twigs near the analysed section of the main stem with a pressure chamber (Model 1000 Pressure Chamber; PMS Instrument Co., Corvallis, OR, USA). These Ψ measurements were assumed to reflect the tension in the xylem of the main axis as a result of low transpiration under laboratory settings.

### Hydraulic vulnerability analysis

Hydraulic vulnerability to drought‐induced embolism was analysed using either the benchtop drying technique with subsequent measurement of flow before and after flushing of embolism (Sperry *et al*., 1988) or saturation under partial vacuum (Hietz *et al*., 2008), or the Cavitron technique (Cochard, 2002), which allowed simultaneous application of water tension and measurement of flow (Table 1).

To avoid common artefacts in hydraulic techniques (e.g. Choat *et al*., 2010; Cochard *et al*., 2013), angiosperm species were only analysed with the centrifuge method when their maximum vessel length was smaller than the rotor diameter (*Amelanchier ovalis*, 19.7 ± 2.5 cm; *Berberis vulgaris*, 27.2 ± 2.5 cm; *Lonicera xylosteum*, 26.6 ± 4.5 cm; *Viburnum lantana*, 17.2 ± 4.3 cm; rotor diameter, 28 cm). Conifers were measured using a modified centrifuge method (Beikircher *et al*., 2010). Long‐vesselled angiosperm species were analysed via benchtop drying.

All sample segments were excised at least 50 cm from the branch's basal end and sequentially cut back, allowing for tension release before the final cutting and subsequent measurements. Samples from species that were collected under tension during the day (shrub species, apple cultivars) were re‐cut by at least twice their mean maximum vessel length to remove potentially introduced embolism in the analysed segments. Thus, we expect that cutting artefacts (Wheeler *et al*., 2013; Torres‐Ruiz *et al*., 2015) can be largely excluded from our analyses, although we note that despite these precautions, some embolism may have been introduced in some longer‐vesselled species measured with the benchtop drying technique.

We fitted the reparameterized Weibull function (Ogle *et al*., 2009) to vulnerability data using the fitplc package (Duursma, 2014) in R version 3.1.1 (R Core Team, 2014), except where hydraulic parameters were previously published. Hydraulic P_50_ was defined as the Ψ at 50% loss of conductivity, which coincides with the inflection point (i.e. the steepest part of the curve) of typical S‐shaped vulnerability curves.

Vulnerability curves obtained with the benchtop drying technique were fitted to pooled data across up to 127 measurements per species per cultivar, owing to the destructive nature of the technique. By contrast, the Cavitron technique allowed for curve‐fitting of three to five individual samples per species and subsequent averaging of computed hydraulic parameters.

### AE analysis

Acoustic emission was monitored with either a PCI‐based system (PAC‐125 PCI‐2 AE System, 18‐bit A/D, 40 MHz) with 150 kHz resonant sensors (R15) and 20/40/60 dB external preamplifiers set to 40 dB, or a USB‐based system (1283 USB AE node, 18‐bit A/D, 20 MHz) with 150 kHz resonant sensors (PK15I, 26 dB integrated preamplifiers; all components from Physical Acoustics Corp., Wolfegg, Germany). Peak definition time, hit definition time, and detection threshold were set to 100–200 μs, 300–400 μs and 35–45 dB, respectively.

Sensors were attached directly to exposed xylem of the main (branch) stem using spring‐loaded clamps (Wolfcraft, Kempenich, Germany), where a small section (1–2 cm^2^) of bark had been removed and covered with silicone grease (Wacker, Burghausen, Germany; or RS Components Ltd, Corby, UK) to improve acoustic coupling and prevent local water loss. AE was recorded from the xylem of dehydrating branches using AEWin for PCI2 version E3.50 (PCI system) or AEWin for USB version E3.35 (USB system; both systems: Physical Acoustics Corp.), respectively, and Ψ was determined periodically. AE with an amplitude of 45 dB or higher was used for analysis in R version 3.1.1 (R Core Team, 2014).

**Figure 1:**
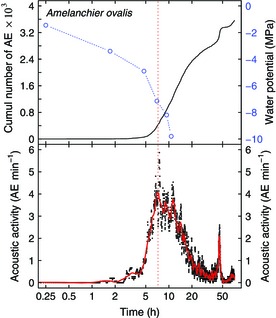
Example graph illustrating the relationship of cumulative acoustic emission (AE) counts and acoustic activity. Upper panel, curve of total cumulative AE (solid curve), water potential (blue open circles) and linear interpolation (dashed blue line) vs time. Lower panel, raw (black squares) and filtered acoustic activity (solid red curve). The red dotted vertical line indicates the timing of maximum rate and Ψ at maximum AE activity (P_maxrate_). Mean SE between parallel water potential measurements was ≤ 0.2 MPa. Note that time is presented on a logarithmic scale.

**Figure 2:**
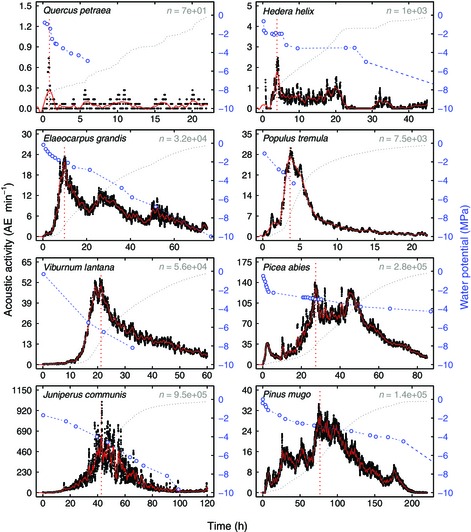
Representative example graphs illustrating the progression of acoustic activity during dehydration in eight species: raw (black circles) and filtered acoustic activity (red solid curves), timing of maximum rate (red dotted vertical lines), water potential data (blue open circles) and linear interpolation (blue dashed lines). Grey dotted lines illustrate corresponding cumulative acoustic emission (AE) curves (linear scale) vs time, whereas the total number of recorded AE (*n*) is given in the top right corner. Mean SE between parallel water potential measurements was ≤ 0.3 MPa in all species.

Acoustic activity was calculated as AE min^−1^ (averaged across 15 min at 1 min increments). Maximum peaks of activity were determined by fitting the Savitzky–Golay smoothing function (function *sgolayfilt* in R; Savitzky & Golay, 1964) to emission rates with a filter order of five, thus reducing variation but preserving the original shape of our data. Finally, the Ψ at maximum AE activity (P_maxrate_) was estimated by linear interpolation between the nearest previous and following Ψ measurements (Figs 1, 2), whereby interpolated Ψ was within 0.3 MPa of the nearest measured Ψ.

### Statistics

Hydraulic P_50_ and P_maxrate_ were tested for least‐squares linear correlation using standard statistical methods (function lm) and model diagnostics (analysis of residuals, leverage, and Cook's distance) in R version 3.1.1 (R Core Team, 2014). All tests were performed at a probability level of *P *<* *0.05.

Confidence intervals (95% CI) for P_50_ obtained from pooled data (benchtop drying technique) were calculated by fitting vulnerability curves to resampled data (999 bootstrap replicates). For all other data, CIs were calculated from the sample mean and SE (Whitley & Ball, 2002):

(Eqn 1) CI=sample mean±1.96SE

where CIs are the upper and lower confidence intervals, respectively. SE values, and therefore CIs, were not computed when *n *<* *3. Values are given as mean, lower CI, and upper CI.

## Results

**Figure 3:**
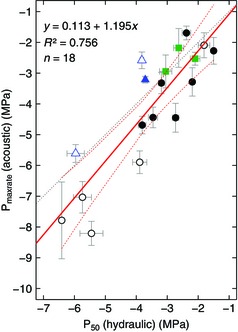
Linear correlation of 50% loss of hydraulic conductivity (P_50_) and the water potential (Ψ) at maximum acoustic emission (AE) activity (P_maxrate_) across 16 species and three crop cultivars of trees (closed symbols) and shrubs or vines (open symbols) of temperate angiosperms (black circles), tropical angiosperms (green squares) and gymnosperms (blue triangles), respectively. Lines indicate the least‐squares linear regression (red solid) and 95% confidence intervals (red dashed), and a 1 : 1 relationship (dotted). Whiskers show ± 1 SE of sample means.

We found a linear correlation (*R* ^2^ = 0.76) between hydraulically measured P_50_ and P_maxrate_ across all studied species, with a correlation coefficient of 1.19 (*P *<* *0.001) and an intercept of −0.11 (*P *≥* *0.05; Fig. 3). Correlation parameters and significance levels changed only minimally after removal of the most influential observations (high leverage and Cook's distance, respectively) or of one species where *n *=* *2, so all data points were included in the analysis.

Hydraulically measured P_50_ ranged between −1.5 and −6.4 MPa across all species, with a mean P_50_ of −3.5 ± 0.3 MPa (Fig. 3; Table 2). As expected, as a result of their habitat, shrub species from the dry forest showed the highest resistance to drought‐induced embolism among the species studied.

**Table 2:** Hydraulic vulnerability (P_50_) and water potential at maximum acoustic activity (P_maxrate_)

Species	P_50_	CI	*n*	P_maxrate_	CI	*n*
Angiosperms						
*Amelanchier ovalis*	−5.45	−6.18, −4.72	5	−8.21	−8.60, −7.82	6
*Berberis vulgaris*	−5.74	−6.31, −5.17	4	−7.03	−7.55, −6.52	5
*Dysoxylum papuanum*	−2.63	−2.97, −2.34	(21)	−2.18	−2.81, −1.55	3
*Elaeocarpus grandis*	−3.05	−3.41, −2.63	(21)	−2.94	−3.48, −2.41	3
*Hedera helix*	−1.81	−2.19, −1.51	(18)	−2.09	−2.50, −1.69	3
*Lonicera xylosteum*	−3.89	−4.34, −3.44	5	−5.90	−6.26, −5.53	5
*Malus domestica* var. *Braeburn*	−3.46	−3.75, −3.17	(49)	−4.44	−4.77, −4.11	4
*Malus domestica* var. *Golden Delicious*	−3.81	−4.05, −3.57	(46)	−4.69	−4.97, −4.40	5
*Malus domestica* var. *Red Delicious*	−2.73	−2.93, −2.53	(40)	−4.45	−4.92, −3.98	4
*Populus alba*	−1.50	−1.60, −1.39	(92)	−2.27	−2.71, −1.84	5
*Populus tremula*	−2.19	−2.33, −2.04	(56)	−3.29	−3.75, −2.82	8
*Quercus petraea*	−2.38	−2.64, −2.10	(35)	−1.69	−1.92, −1.47	3
*Sorbus aucuparia*	−3.19	−3.36, −3.01	(33)	−3.32	−3.67, −2.97	3
*Syzygium sayeri*	−2.10	−2.47, −1.87	(19)	−2.53	−	2
*Viburnum lantana*	−6.41	−6.55, −6.27	4	−7.79	−9.03, −6.54	4
Gymnosperms						
*Juniperus communis*	−5.96	−6.51, −5.41	5	−5.61	−5.90, −5.32	13
*Picea abies*	−3.71	−3.81, −3.59	(127)	−3.21	−3.32, −3.11	3
*Pinus mugo*	−3.82	−3.94, −3.70	3	−2.58	−2.85, −2.31	5

Mean, 95% confidence interval (CI, lower and upper), and number of samples. Values in parentheses are the number of individual measurements used for pooled curve‐fitting. Confidence intervals were not calculated when *n *<* *3.

**Figure 4:**
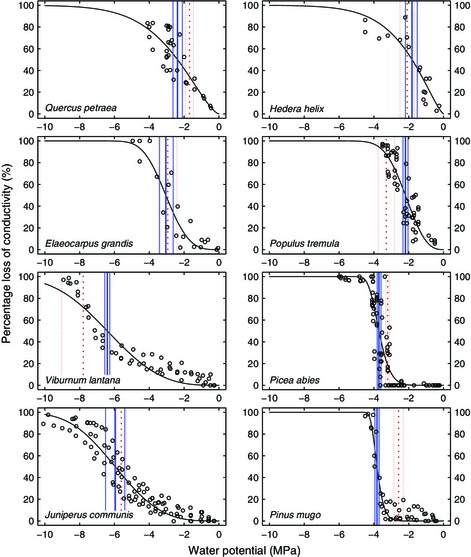
Representative example graphs illustrating the relationship of 50% loss of hydraulic conductivity (P_50_) and the water potential (Ψ) at maximum acoustic emission (AE) activity (P_maxrate_) in hydraulic vulnerability curves in eight species according to Fig. 2: hydraulic measurements (open circles) and fitted Weibull curves (black solid lines). Blue solid lines indicate P_50_ (thick lines) and 95% confidence intervals (CIs, thin lines), and red dotted lines indicate P_maxrate_ (thick lines) and 95% CI (thin lines).

P_maxrate_ was between −1.7 and −8.2 MPa across all species (average, −4.1 ± 0.5 MPa), and was more negative than P_50_ in all but three angiosperms, but less negative than P_50_ in the three conifer species (Figs 3, 4). A comparison of the analysis of total cumulative AE vs AE activity is presented in Fig. 1.

Example graphs illustrating the progression of AE activity and analysis of maximum peaks for ring‐porous angiosperms (*Quercus petraea*, temperate tree; *Hedera helix*, temperate vine, semi‐ring‐porous), diffuse‐porous angiosperms (*Elaeocarpus grandis*, tropical tree; *Populus tremula*, temperate tree; *V. lantana*, temperate shrub) and conifers (*Picea abies*, temperate tree; *Juniperus communis*, temperate tree or shrub; *Pinus mugo*, temperate shrub) are shown in Fig. 2.

## Discussion

We showed that a species’ hydraulic vulnerability can be estimated by determining the temporal density of acoustic emissions during increasing amounts of drought stress. As hypothesized, the maximum acoustic activity (P_maxrate_) was reached at a Ψ corresponding to the Ψ at or near the steepest point of hydraulic vulnerability curves in most species, and therefore was correlated to hydraulically measured P_50_.

Our analysis covers a broad range of P_50_ spanning 4.9 MPa. All hydraulic vulnerability curves were S‐shaped, and an exemplary selection is presented in Fig. 4. The most vulnerable species were *P. tremula* and the vine *H. helix* (P_50_ of −1.5 and −1.8 MPa, respectively), and *V. lantana* and *J. communis* were the least vulnerable species (P_50_ of −6.4 and −6.0 MPa).

Acoustic emission testing offers a less laborious and noninvasive way to answer plant hydraulic questions in the laboratory and *in situ*. The method enables monitoring of the occurrence and timing of cavitation events in plants noninvasively, and has been successfully used to estimate plant hydraulic vulnerability in some species (Lo Gullo & Salleo, 1993; Hacke & Sauter, 1995; Hacke *et al*., 2000; Rosner *et al*., 2006; Vergeynst *et al*., 2013, 2014a).

In many species, plots of cumulative AE vs time first resemble an exponential‐sigmoid curve similar to S‐shaped hydraulic vulnerability curves, but later deviate from that shape and continue rising despite reaching complete hydraulic failure (Rosner, 2015; Figs 1, 2). This additional AE occurring at late stages of dehydration often cannot be distinguished from AE that originates in conducting elements, and thus exclusion from quantitative vulnerability analysis remains difficult (Rosner, 2012). Unless this emission can be accounted for, the higher total number of recorded signals should result in lower estimated hydraulic parameters, such as P_12_, P_50_ and P_88_. Authors have thus been looking for ways to minimize the effects of noise on vulnerability analysis, for example by manually setting end points of recording (Hacke & Sauter, 1996; Kikuta *et al*., 1997; Salleo *et al*., 2001) or by looking at amplitudes or energy properties rather than signal amplitudes (Rosner *et al*., 2006, 2009; Mayr & Rosner, 2011; Johnson *et al*., 2012), but a universal, automatable procedure is still lacking.

Our method offers a convenient way to avoid the effect of these late‐stage signals by looking at acoustic activity over time, rather than cumulative emissions per Ψ step. This approach allowed us to determine the Ψ at which most cavitation occurred within a short time‐frame (P_maxrate_) during dehydration, while minimizing the influence of late‐stage signals below the physiologically relevant range in Ψ (Figs 1, 2). P_50_ values, the points at which most embolism forms per Ψ decrement, were thus correctly estimated using P_maxrate_.

Interestingly, P_maxrate_ values were more negative than P_50_ in all but three angiosperm species, indicating that the highest density of cavitation events occurs at Ψ values slightly lower than hydraulic P_50_. By contrast, all conifer species revealed P_maxrate_ to be higher than P_50_ (Figs 3, 4; Table 2). We believe the differences between P_50_ and P_maxrate_, and the differences between angiosperms and gymnosperms, result from several contributing factors.

First, AE provides information about cavitation events and not their effects on hydraulic conductivity. This distinction is especially important in angiosperms, where conduits in a xylem cross‐section are heterogeneous in size and differ in their hydraulic conductivity, as cavitation in both a small conduit and a much larger vessel is expected to result in one AE causing embolism (Jackson & Grace, 1996). Emissions might also be caused by nanobubble formation, which may or may not expand to form embolism (Schenk *et al*., 2015). Wide conduits also cavitate at more moderate Ψ than smaller conduits (Tyree & Sperry, 1989; Tyree & Zimmermann, 2002), so we expect that more relative conductivity is lost in angiosperms during early cavitation, while later stages should produce more signals causing considerably lower hydraulic decline. In conifers, the high number of small, vulnerable and hydraulically less important tracheids in latewood and compression wood (Mayr & Cochard, 2003) may have caused high acoustic activity before P_50_ was reached.

Second, AE can originate in both conducting and nonconducting tissues, such as fibre tracheids, ray cells and sclerenchyma (Tyree & Dixon, 1986; Jackson & Grace, 1996; Kikuta, 2003; Kikuta & Richter, 2003), which means that not every individual signal necessarily represents an increase in xylem embolism.

Third, xylem is a heterogeneous medium for sound, and sound propagation velocities vary for air (embolized conduits), water (xylem sap) and solid materials (cell walls). Sound waves are influenced by these factors, resulting in complex acoustic sound conduction and attenuation effects before signals arrive at the sensor, which further complicates analysis (Tyree & Sperry, 1989; Jackson & Grace, 1996; Kikuta *et al*., 1997; Bucur, 2006; Mayr & Rosner, 2011). Such acoustic effects, in combination with the variability of sample anatomy, sample size or geometry, and acoustic coupling of the AE sensor, also influence the total number of recorded AEs (Fig. 2), which adds importance to the use of relative AE counts or rates.

Finally, despite the precautions taken, some artificial embolism may have been introduced in species with longer vessels during sampling or Ψ determination. This would mean that some angiosperm species might be less vulnerable to embolism formation than hydraulically measured, and could explain part of the negative shift in P_maxrate_ (Figs 3, 4). Furthermore, our rate‐based analysis requires careful evaluation when the Ψ decrease is not constant, as AE may simply occur on a shorter timescale for part of the measurement, rather than revealing a higher number of signals per Ψ decrement (e.g. for *Q. petraea* in Fig. 2).

When viewed across a sample set of 18 data points (16 species and three cultivars), the expected linear correlation between P_50_ and P_maxrate_ is clearly present (Fig. 3), but as a result of the varying shift between acoustic and hydraulic parameters (Fig. 4), hydraulic measurements may still be required for precise comparison between samples of similar hydraulic vulnerability. While our study does not allow evaluation of whether variation in P_maxrate_ is a result of real variation in P_50_ or of measurement uncertainty, such covariation between P_50_ and P_maxrate_ deserves testing in further experiments.

We believe that the use of AE rates instead of counts may be a valuable option for noninvasive monitoring of cavitation *in situ*: peaking rates may indicate the timing of considerable drought stress under controlled conditions, whereby possible temperature effects need to be considered. The presented analysis of AE activity allows an estimate of the hydraulic P_50_ of a study species or population and can provide new insights into the timing of drought stress and embolism formation.
